# Supplementary material for: The Chloroplast of Chlamydomonas reinhardtii as a Testbed for Engineering Nitrogen Fixation into Plants
Source: Int J Mol Sci. 2021 Aug 16;22(16):8806. doi: 10.3390/ijms22168806 (PMC8395883; doi:10.3390/ijms22168806)
Supplement: Supplementary file 1 [file ijms-22-08806-s001.zip › ijms-1286091-supplementary.pdf]

International Journal of
Molecular Sciences

MDPI

Review

The Chloroplast of *Chlamydomonas reinhardtii* as a Testbed for Engineering Nitrogen Fixation into Plants

Marco Larrea-Álvarez[1] and Saul Purton[2,*]

[1] School of Biological Sciences and Engineering. Yachay-Tech University Hacienda San José, Urcuquí-Imbabura 100650, Ecuador; malarrea@yachaytech.edu.ec
[2] Algal Research Group, Institute of Structural and Molecular Biology, University College London, Gower Street, London WC1E 6BT, UK; s.purton@ucl.ac.uk
* Correspondence: s.purton@ucl.ac.uk

Supplementary Methods

1. Bioinformatic analyses

We performed an *in silico* study to look for potential *nifU* and *nifS* homologs in the *C. reinhardtii* nuclear genome. Similarity searches, using *K. oxytoca* NifU (WP_109213089.1) and NifS (CAB1215886.1) protein sequences as queries, were performed using PSI-Blast [1] against RefSeq databases; searches were limited to *C. reinhardtii* and excluded partial and predicted sequences. The NCBI CD-search tool (https://www.ncbi.nlm.nih.gov/Structure/cdd/wrpsb.cgi) and the ChloroP 1.1 predictor server [2] were used for conserve domain searching and subcellular localization prediction, respectively. Multiple sequence alignment was carried out using the CLUSTALW program [3]. Percentages of similarities between ChlL (ASF83651.1), NifH (VTS50234.1) and AnfH (SFW98605.1), were produced using the BLAST tool (https://blast.ncbi.nlm.nih.gov) [4].

2. pNifV chloroplast expression vector

The gene sequence encoding the homocitrate NifV from *K. oxytoca* (WP 023322951.1) was codon-optimised according to the codon bias found in the *C. reinhardtii* plastome (sequence given below) and cloned into the pSRSapI [5] vector using SapI and SphI sites. The resulting plasmid was named pNifV.

>crnifV: 1,176 bp

```
ATGATGGGTCGTGTATTAATTAACGATACTACTTTACGTGACGGTGAACAATCTCCAGGTGTAG
CTTTCCAAGCTTCAGAAAAAATTGCTATTGCTGAAGCTTTATACGCTGCTGGTGTTGAAGCTAT
GGAAGTAGGTACTCCAGCTATGGGTGAAGAAGAATGTGCTCGTATTCGTCAAGTTCGTCGTCAA
TTACCAGGTGCTACTTTAATGACATGGTGTCGTATGCAAGCTGGTGAAATTCGTCAATCAGCTG
ATTTAGGTATGGACTGGGTTGATATTTCTATTCCAGCTTCAGACAAATTACGTCAATACAAATT
ACGTGAAGGTTTACCATTATTATTAGAACGTTTAGCTGCTTTAATTCACTTAGCTCACACTTTA
GGTTTAAAAGTATGTATTGGTTGTGAAGATGCTTCACGTGCTTCTGATGCTACTTTACAAGATA
TTGCTCGTTTAGCTCGTGAAGCTGGTGCTACTCGTTTACGTTACGCTGATACTGTAGGTATTTT
AGATCCATTTACTACAGCTGCTCAAATTGCTGCTTTACGTCGTGTTTGGCCAGGTGAATTAGAA
ATGCACGCTCACAACGATTTAGGTTTAGCTACTGCTAACACTTTAGCTGCTGTTCGTGCTGGTG
CTACTTCTGTAAACACAACAGTATTAGGTTTAGGTGAACGTGCTGGTAACGCTGCTTTAGAAAC
AGTAGCTTTAGGTTTAGAACGTTGTTTAGAAGTTAAAACAGGTGTTCGTTTCACTGCTTTACCA
GCTTTATGTGAACAAGTAGCTTTAGCTACTCGTCGTCCAGTAGATCCACAACAACCATTAGTAG
GTGAATTAGTATTTACTCACGAATCAGGTGTTCACGTAGCTGCTTTATTACGTGACAGTGAATC
ATACCAAGCTATTGATCCAGCTTTATTAGGTCGTGGTTACCGTTTAGTTTTAGGTAAACACTCT
GGTCGTCAAGCTGTTAACGGTGTTTTTGACCGTATGGGTTACCACTTAACATCAGCTCAAATTG
ACCAATTATTACCAGCTTTACGTCGTTTCGCTGAAAACGGTAAACGTTCTCCACGTGATGATGA
ATTAGCTGCTATTTACCACGCTTTATGTTCAGCTGAAACTTTACAAGCTCGTGGTTACCCATAC
GATGTTCCAGATTACGCTTAATAA
```

References

1. Altschul, S; Madden, T; Schäffer, A; Zhang, J; Zhang, Z; Miller. W; et al. Gapped BLAST and PSI-BLAST: a new generation of protein database search programs. *Nucleic Acids Res* **1997,** 25: 3389–402.
2. Emanuelsson O, Nielsen H, von Heijne G. ChloroP, a neural network-based method for predicting chloroplast transit peptides and their cleavage sites. *Protein Sci* **1999**, 8: 978–984.
3. Thompson, D; Higgins, D; Gibson, T. CLUSTAL W: improving the sensitivity of progressive multiple sequence alignment through sequence weighting, position-specific gap penalties and weight matrix choice. *Nucleic Acids Res* **1994,** 11:4673–80.
4. Camacho, C; Coulouris, G; Avagyan, V; Ma, N; Papadopoulos, J; Bealer, K; et al. BLAST+: architecture and applications. *BMC Bioinformatics*, **2009**, 10: 421.
5. Young, R; Purton, S. Cytosine deaminase as a negative selectable marker for the microalgal chloroplast: a strategy for the isolation of nuclear mutations that affect chloroplast gene expression. *Plant J*, **2014**, 80: 915–925.
